# First autochthonous cases of Oropouche fever in Southern Brazil: A case series in a non-endemic area, Santa Catarina, 2024

**DOI:** 10.1590/S2237-96222026v35e20250864.en

**Published:** 2026-04-24

**Authors:** Renan Novaes Pinto, Benvindo Joãozinho Sá, Ícaro Gonçalves Santos, Vinicius Pereira Feijó, Ivânia da Costa Folster, Renata Zeferino Marques, Leila Catiani Pedrini Eyng, Natália de Andrade Cabral Marchi, Nilceia das Graças Garcia de Souza, Roseleti Martendal, Fernanda Aragona da Costa, Alessandro Igor da Silva Lopes, Morgana de Freitas Caraciolo, Daniel Garkauskas Ramos, Livia Carla Vinhal Frutuoso, Danielle Cristine Castanha da Silva, Aroldo José Borges Carneiro

**Affiliations:** 1 Ministério da Saúde, Programa de Treinamento em Epidemiologia Aplicada aos Serviços do SUS, Brasília, DF, Brazil Ministério da Saúde Programa de Treinamento em Epidemiologia Aplicada aos Serviços do SUS Brasília DF Brazil; 2 Ministério da Saúde, Departamento de Doenças Transmissíveis, Brasília, Distrito Federal, Brazil Ministério da Saúde Departamento de Doenças Transmissíveis Brasília Distrito Federal Brazil; 3 Secretaria de Estado da Saúde de Santa Catarina, Florianópolis, Santa Catarina, SC, Brazil Secretaria de Estado da Saúde de Santa Catarina Florianópolis Santa Catarina, SC Brazil; 4 Secretaria Municipal de Saúde de Botuverá, Botuverá, Santa Catarina, SC, Brazil Secretaria Municipal de Saúde de Botuverá Botuverá Santa Catarina, SC Brazil; 5 Secretaria Municipal de Saúde de Brusque, Brusque, Santa Catarina, SC, Brazil Secretaria Municipal de Saúde de Brusque Brusque Santa Catarina, SC Brazil; 6 Secretaria Municipal de Saúde de Guabiruba, Guabiruba, Santa Catarina, SC, Brazil Secretaria Municipal de Saúde de Guabiruba Guabiruba Santa Catarina, SC Brazil; 7 Secretaria Municipal de Saúde de Luiz Alves, Luiz Alves, Santa Catarina, SC, Brazil Secretaria Municipal de Saúde de Luiz Alves Luiz Alves Santa Catarina, SC Brazil; 8 Secretaria Municipal de Saúde de Ilhota, Ilhota, Santa Catarina, SC, Brazil Secretaria Municipal de Saúde de Ilhota Ilhota Santa Catarina, SC Brazil

**Keywords:** Oropouche Virus Infection, Arbovirus Infections, Disease Outbreaks, Epidemiological Investigation, Epidemiology, Descriptive, Infección por el Virus Oropouche, Infecciones por Arbovirus, Brotes de Enfermedades, Investigación Epidemiológica, Epidemiología Descriptiva

## Abstract

**Objective:**

To describe time, person, and place of the first Oropouche fever confirmed in the state of Santa Catarina, evaluating their autochthonism.

**Methods:**

A descriptive case series study conducted with residents of five Santa Catarina municipalities, who tested positive for Oropouche fever in laboratory tests between April 25 and June 12, 2024. Retrospective samples, negative for dengue, and viable for molecular analysis (RT-PCR) were considered. Secondary data collected during epidemiological investigations and information systems were used. Statistical analysis was performed on SPSS 18.0. Autochthonous cases were defined as those with no travel history 15 days prior to symptoms onset.

**Results:**

Of the 41 eligible cases, 33 participated in the study. Most were female (63.6%), white (63.6%) and of economically active age (78.8%). Most frequent symptoms included headache (93.9%), fever (90.9%) and myalgia (81.8%). About half reported recurrence of symptoms after clinical cure. Most reported exposure to waterbodies (57.6%) and areas with natural vegetation (48.5%), as well as frequent presence of the *Culicoides* sp. in homes and workplaces. Most lived and worked near environments conducive to vector proliferation such as agricultural areas with banana cultivation. No case reported travel to other federative units or abroad.

**Conclusion:**

Oropouche cases in Santa Catarina presented a clinical-epidemiological profile compatible with previous descriptions. Unprecedented detection of the Oropouche virus in the region reinforces the need for timely diagnosis and integrated surveillance for emerging and reemerging arboviruses in Brazil.

## Resumen

**Objetivo:**

Describir en tiempo, persona y lugar los primeros casos de la fiebre de Oropouche confirmados en Santa Catarina (Brasil) para evaluar su autoctonía.

**Métodos:**

Estudio descriptivo de series de casos realizado con residentes de cinco municipios de Santa Catarina, detectados por pruebas de laboratorio para la fiebre de Oropouche entre el 25 de abril y el 12 de junio de 2024. Se consideraron muestras retrospectivas, negativas para dengue y viables para análisis molecular (RT-PCR). Se utilizaron datos secundarios recopilados durante los estudios epidemiológicos y de sistemas de información. Los análisis se realizaron con estadística descriptiva. Los casos autóctonos fueron aquellos con ausencia de desplazamiento 15 días antes del inicio de los síntomas.

**Resultados:**

De los 41 casos elegibles, participaron 33 en el estudio. La mayoría de los sujetos eran del sexo femenino (63,6%), blancos (63,6%) y económicamente activos (78,8%). Los síntomas más frecuentes fueron cefalea (93,9%), fiebre (90,9%) y mialgia (81,8%). Aproximadamente la mitad informó recurrencia de los síntomas después de la curación clínica. Una gran parte de ellas reportó exposición a cuerpos de agua (57,6%) y áreas con vegetación natural (48,5%), así como presencia frecuente del vector *Culicoides* sp. en hogares y lugares de trabajo. La mayoría vivía y trabajaba cerca de entornos propicios para la proliferación del vector, como las zonas agrícolas con cultivo de banana. No se reportaron casos de desplazamiento a otras unidades de la federación o al exterior.

**Conclusión:**

Los casos de Santa Catarina presentaron un perfil clínico-epidemiológico compatible con las descripciones anteriores. La detección sin precedentes del virus Oropouche en la región destaca la necesidad de diagnósticos adecuados y de vigilancia integrada para las arbovirosis emergentes y reemergentes en Brasil.

**Ethical aspects**

**This research respected the ethical principles, having obtained the following approval data:**

Research Ethics Committee National Research Ethics Commission

Opinion number 7.665.911

Approval date Jun 28, 2025

Certificate of Submission for Ethical Appraisal: 88060725.4.0000.0008

Registration informed consent Exempted.

## Introduction

Oropouche fever is an arbovirus considered endemic to the Amazon region, with outbreaks recorded in other regions of Brazil and Latin America ^1^. Since 2023, Brazil has shown a significant increase in the number of reported Oropouche fever cases, especially in states outside the Amazon ^2^^),(^^3^.

Initially concentrated in states within the Brazilian Legal Amazon, between 2024 and 2025 the disease expanded rapidly to areas considered non-endemic, with explosive outbreaks occurring mainly in the Northeast and Southeast, in states such as Bahia, Ceará, Rio de Janeiro, Minas Gerais, and Espírito Santo ^2^^)-(^^7^.

According to public data from the Ministry of Health, Brazil registered 834 cases in 2023, 13,856 in 2024 and 11,962 until Epidemiological Week 30 of 2025 ^8^.

Oropouche virus (OROV) infection usually manifests in a mild and self-limited form. However, an alert was recently issued due to deaths associated with the disease, as well as confirmation of possible vertical transmission and cases of congenital anomalies and fetal deaths related to the infection ^9^^)-(^^11^.

Occurrence of explosive outbreaks in different Brazilian regions, including non-endemic areas, associated with new epidemiological findings related to deaths and vertical transmission represents an important alert for public health.

Given this context, the unprecedented detection of OROV in Santa Catarina characterizing the first record in Southern Brazil, an area considered non-endemic, motivated an epidemiological investigation to describe the first confirmed cases in the state regarding time, person and place, evaluating their autochthonism. 

## Methods

### Study design

This is a descriptive case series study conducted based on laboratory testing for Oropouche virus (OROV) between April 25 and June 12, 2024, with residents of Botuverá, Brusque, Guabiruba, Ilhota and Luiz Alves-municipalities concentrating the confirmed cases, until that moment, in Santa Catarina.

### Background

In early 2024, Botuverá, in Santa Catarina, recorded an unusual increase in suspected cases of dengue fever, an atypical epidemiological scenario compared to the municipality’s history. However, most samples showed negative laboratory results for dengue fever, and the entomological investigation indicated low infestation of *Aedes aegypti* in the municipality. Given this scenario, coupled with negative results in diagnoses for other investigated diseases, a clinical profile compatible with arboviruses and Oropouche fever cases identified during the same period in non-endemic areas for the disease in Brazil ^2^, the hypothesis that it was Oropouche fever began to be considered. Based on laboratory analysis of five samples from symptomatic patients in the acute phase, during an opportune period for molecular diagnosis, the first three positive OROV cases were detected in Santa Catarina on April 25, 2024. 

Based on these detections and given the atypical epidemiological scenario being observed in Botuverá, the Central Public Health Laboratory of Santa Catarina conducted retrospective analyses for OROV on samples from the municipality that tested negative for dengue, Zika, and chikungunya, stored at the institution. Based on new OROV detections in retrospective analyses, this testing strategy was expanded to include clinical material from nearby municipalities. As a result, the virus was detected in dozens of samples, confirming the occurrence of an outbreak in the state during the first half of 2024.

Santa Catarina is one of the 27 federative units of Brazil, located in the Southern region. It borders Paraná to the north and Rio Grande do Sul to the south, is bathed by the Atlantic Ocean to the east, and has an international border with Argentina to the west. It has a population of 7,610,361 inhabitants distributed across a territorial area of 95,730.690 km², with a Human Development Index (HDI) of 0.792, classified as high ^12^. The state’s 295 municipalities are distributed across eight health macro-regions and 17 health regions ^13^. 

### Participants

Between April 50 and June 12, 2024, Santa Catarina registered 50 cases of Oropouche fever. Of these, 41 were considered eligible according to the following criteria: (i) residence in one of the five municipalities selected for the study-Botuverá, Brusque, Guabiruba, Ilhota or Luiz Alves-; (ii) initial notification as a suspected case of dengue; (iii) sample collection in the acute phase for molecular diagnosis at the Central Public Health Laboratory; and (iv) undetectable result for the dengue virus and detectable for OROV.

Cases were considered autochthonous in Santa Catarina when no history of travel to other states or countries was identified in the 15 days prior to symptom onset, considering the incubation period of Oropouche fever estimated to be 3 to 10 days ^1^^),(^^9^^),(^^14^. 

### Variables

The instrument for data collection, applied by the epidemiological investigation teams in the territories, included sociodemographic variables (place of residence; gender; age; ongoing pregnancy; race/skin-color; schooling level; professional activity and workplace), clinical-epidemiological variables (date of symptom onset; signs and symptoms; duration of symptoms; symptom recurrence; other symptomatic individuals in the residence or neighborhood; chronic disease or condition; travel in the 15-day period before symptom onset and interval between symptom onset and the time of investigation), related to habits that could favor disease exposure (exposure to environments with potential presence of vectors; use of individual or collective protective measures/equipment; recurrence of vector bites) and related to the environmental characteristics of the residential and workplace locations (territorial zone; proximity to areas with waterbodies, natural vegetation, agricultural cultivation and deforestation; type of agricultural cultivation reported; presence of insect vectors of arboviruses). 

Professional activities were classified according to the major groups of the Brazilian Classification of Occupations. For individuals who reported working in more than one location, data was collected for each location specifically.

### Data sources and measurement

Anonymized secondary data, provided by the General Coordination of Arbovirus Surveillance of the Ministry of Health and the Santa Catarina State Department of Health, were used. These data were obtained via the information systems used by surveillance-SINAN and Laboratory Environment Manager (GAL)-and from field investigations, recorded in a standardized and systematized manner on a semi-structured questionnaire applied by the epidemiological surveillance teams of the territories involved, as a health surveillance action, with support from the advanced level of the Training Program in Applied Epidemiology for the Unified Health System Services (EpiSUS-Avançado).

### Bias

To minimize recall bias among interview participants, priority was given to using information recorded in municipal health information systems and records for the variables of symptom onset date and reported signs and symptoms.

### Study size

Analysis was conducted on 33 cases of Oropouche fever, out of the 50 detected in Santa Catarina from April 25 to June 12, 2024.

### Statistical methods

Descriptive statistics were used, with calculations of absolute and relative frequency measures, as well as measures of central tendency and dispersion, carried out on Microsoft Excel 365.

A histogram of confirmed Oropouche fever cases was created based on the date of symptom onset and plotted by epidemiological week. Cases were categorized into those that were included and those that were not included in the study.

Spatial distribution maps of the cases were created on QGIS 3.34.4, using anonymized latitude and longitude data for georeferencing the residential and workplace locations, and vector data for municipality, state, and country layers, provided by the Brazilian Institute of Geography and Statistics (IBGE), under the UTM projection, zone 22J, SIRGAS 2000 datum.

## Results

Between April 25 and June 12, 2024, Santa Catarina registered 50 cases of Oropouche fever. Of these, 41 met the eligibility criteria among which 33 agreed to participate in the interviews conducted by the local epidemiological surveillance teams, and were therefore included in the analysis. Eight individuals refused to participate. Regarding symptom onset date, most included cases fell between epidemiological weeks 11 and 20 of 2024, with case peaks occurring in weeks 13 and 19 (Figure 1). 

**Figure 1:**
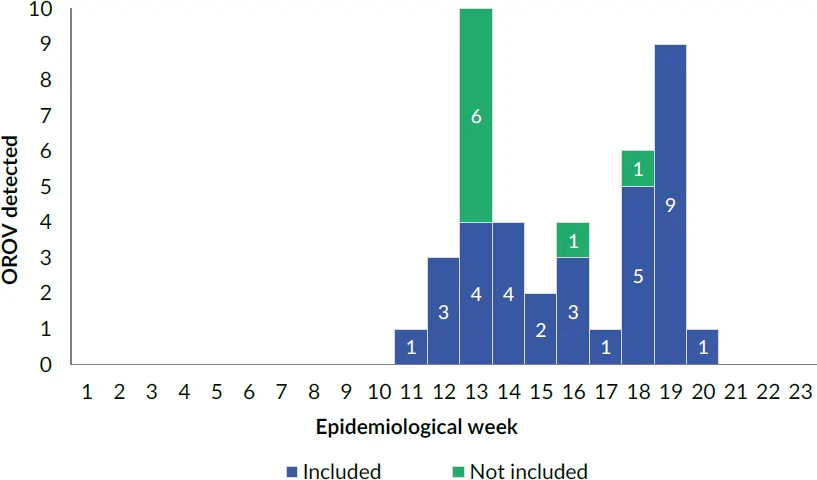
Number of confirmed Oropouche fever cases per epidemiological week from symptom onset, according to cases included (n=33) and not included (n=8) in the study. Santa Catarina, 2024 (n=41)

Of the 33 study participants, most lived in Luiz Alves (12; 36.4%) and Botuverá (10; 30.3%) municipalities. Most patients were female (21; 63.6%), self-declared white (21; 63.6%) with ages from 20 to 59 years (26; 78.8%). Median age of the interviewees was 38 years (13-66 years). No pregnant women featured among the interviewed cases. As for education, most participants had incomplete middle school (13; 39.4%) followed by incomplete high school (11; 33.3%), totaling over half of the interviewees (24; 72.7%). Most participants (25; 75.8%) reported having at least one employment contract, predominantly in the category of industrial goods and services (13; 52.0%). Of the eight interviewees without a professional activity, four were retirees (50.0%) (Table 1).

**Table 1:** Sociodemographic characteristics of Oropouche fever cases. Santa Catarina, 2024 (n=33)

Variables	n (%)
**Municipality of residence**	
Luiz Alves	12 (36.4)
Botuverá	10 (30.3)
Brusque	5 (15.2)
Ilhota	5 (15.2)
Guabiruba	1 (3.0)
**Gender**	
Female	21 (63.6)
Male	12 (36.4)
**Age group (years)**	
10-14	1 (3.0)
15-19	2 (6.1)
20-29	8 (24.2)
30-39	7 (21.2)
40-49	6 (18.2)
50-59	5 (15.2)
60-69	4 (12.1)
**Race/skin-color**	
White	21 (63.6)
Mixed-race	10 (30.3)
Yellow	1 (3.0)
Indigenous	1 (3.0)
**Schooling level**	
Incomplete middle school	13 (39.4)
Complete middle school	1 (3.0)
Incomplete high school	11 (33.3)
Complete high school	2 (6.1)
Incomplete higher education	2 (6.1)
Complete higher education and higher	4 (12.1)
**Professional activity**	
**Employed (Large group from the Brazilian Classification of Occupations)**	25 (75.8)
Workers of industrial goods and services production	13 (52.0)
Service workers, commerce saleswomen in shops and markets	7 (28.0)
Agricultural, forestry and fisheries workers	2 (8.0)
Administrative services workers	2 (8.0)
Science and arts professionals	1 (4.0)
Senior members of the public authorities and leaders of organizations and companies	1 (4.0)
**No employment**	8 (24.2)
Retired	4 (50.0)
Home caregiver	3 (37.5)
Student	1 (12.5)

Headache (31; 93.9%), fever (30; 90.9%), myalgia (27; 81.8%), loss of appetite (26; 78.8%), fatigue/prostration (25; 75.8%), and arthralgia (24; 72.7%) were the signs and symptoms most frequently reported (Figure 2). Median duration of symptoms was 14 days, with a minimum of two days and a maximum of 37 days. Of the 33 participants, 17 (51.5%) reported experiencing similar symptoms up to 15 days after clinical cure, mainly headache (11; 64.7%), weakness (7; 41.2%) and myalgia (5; 29.4%). We identified no record of severe cases.

**Figure 2:**
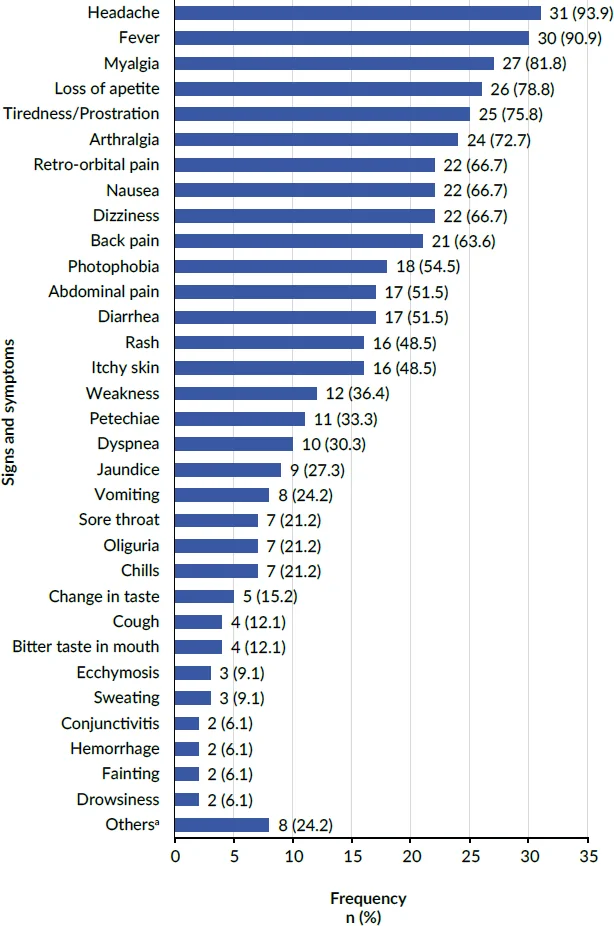
Characterization of Oropouche fever cases according to signs and symptoms. Santa Catarina, 2024 (n=33) ^a^ Loss of consciousness; loss of taste; burning sensation in the skin; trembling hands; confusion; ear blockage (autophony); dehydration and urine odor.

Notably, 18 (54.5%) participants reported that at least one other symptomatic resident in the household had been ill during the same period as them, and 17 (51.5%) mentioned knowing of a neighbor with similar symptoms during the same period.

Of the 33 participants, 19 (57.6%) reported having some chronic disease or condition, with high blood pressure (8; 42.1%) and respiratory diseases (4; 21.1%) being the most frequently reported.

All trips mentioned by the interviewees, in the period of up to 15 days prior to symptom onset, occurred between municipalities in Santa Catarina, with no records of trips to other states or abroad.

Regarding exposure to environments potentially related to the presence of vectors, 19 (57.6%) respondents reported frequently traveling to areas with bodies of water. For areas with natural vegetation, 16 (48.5%) reported this type of exposure.

All interviewees reported employing some protective measure to prevent contact with midges: 29 (87.9%) reported using long clothing, 28 (84.8%) used repellents, and 24 (72.7%) used both measures.

Recurrence of midge bites was reported by 30 (90.9%) participants, whereas 28 (84.8%) mentioned recurrence of mosquito bites.

Regarding environment, 33 residential locations and 27 workplaces were characterized. Most residential locations were located in urban areas (19; 57.6%), as were the workplaces (17; 63.0%) (Table 2).

**Table 2:** Characterization of the home (n=33) and work (n=27) environments of the Oropouche fever cases. Santa Catarina, 2024

Variables	Environment	
	Home (N=33)	Work (N=27)
	n (%)	n (%)
**Area**		
Urban	19 (57.6)	17 (63.0)
Rural	14 (42.4)	10 (37.0)
**Waterbodies**		
Yes	27 (81.8)	22 (81.5)
Tributary^a^	21 (77.8)	11 (50.0)
Lake	5 (18.5)	1 (4.5)
River	8 (29.6)	12 (54.5)
Pond	-	1 (4.5)
No	6 (18.2)	5 (18.5)
**Natural vegetation**		
Yes	30 (90.9)	22 (81.5)
No	3 (9.1)	5 (18.5)
**Agricultural crops**		
Yes	23 (69.7)	14 (51.9)
Cassava	2 (8.7)	-
Rice	1 (4.3)	1 (7.1)
Banana	13 (56.5)	7 (50.0)
Potato	1 (4.3)	-
Sugarcane	-	1 (7.1)
Grass	2 (8.7)	1 (7.1)
Eucalyptus	1 (4.3)	1 (7.1)
Flowers	-	1 (7.1)
Greenery	10 (43.5)	5 (35.7)
Corn	4 (17.4)	1 (7.1)
Other fruits^b^	3 (13.0)	-
Palm tree	3 (13.0)	1 (7.1)
No	9 (27.3)	12 (44.4)
I do not know	1 (3.0)	1 (3.7)
**Deforestation (last two years)**		
Yes	11 (33.3)	3 (11.1)
No	21 (63.6)	23 (85.2)
I do not know	1 (3.0)	1 (3.7)
**Presence of *Culicoides* (midge)**		
Yes	30 (90.9)	23 (85.2)
No	3 (9.1)	4 (14.8)
**Presence of mosquitoes**		
Yes	29 (87.9)	15 (55.6)
No	4 (12.1)	11 (40.7)
I do not know	-	1 (3.7)

^a^ Stream, brook, creek, canal and spring; ^b^ Orange, strawberry and tangerine

Presence of waterbodies (rivers, tributaries, lakes and ponds) was reported near 27 (81.8%) residences and 22 (81.5%) workplaces. Tributaries were the most frequently mentioned waterbodies near residences, with 21 (77.8%) records, and rivers were the most cited near workplaces, with 12 (54.5%) reports. Proximity to natural vegetation areas was reported for 30 (90.9%) residences and 22 (81.5%) workplaces (Table 2). 

Agricultural cultivation was reported near 23 (69.7%) residences and 14 (51.9%) workplaces. In both contexts, bananas were the most frequently mentioned crop (Table 2). 

No deforestation was reported in the last two years near 21 (63.6%) and 23 (85.2%) workplaces (Table 2).

Presence of *Culicoides* midges was reported in both environments, with 30 (90.9%) records in residential locations and 23 (85.2%) in workplaces. Regarding mosquitoes, 29 (87.9%) reports were recorded in residences and 15 (55.6%) in workplaces (Table 2).

Spatial distribution of the residences and workplaces indicated proximity to waterbodies and/or areas with vegetation cover (Figure 3).

**Figure 3:**
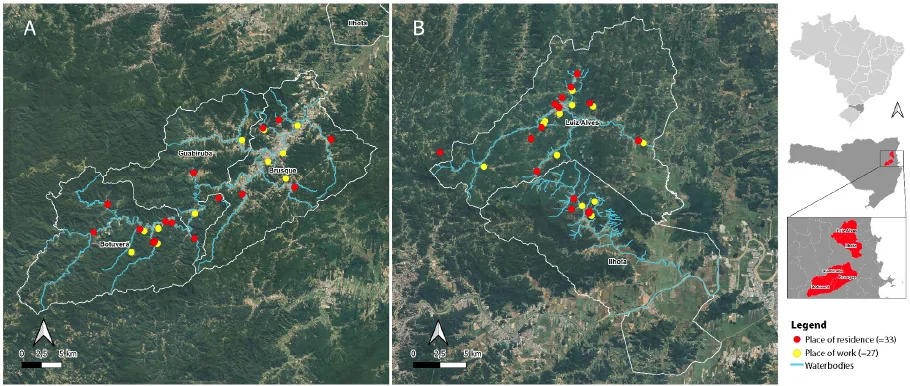
Spatial distribution of places of residence (n=33) and work (n=27) of Oropouche fever cases in Botuverá, Brusque and Guabiruba (A) and in Luiz Alves and Ilhota (B). Santa Catarina, 2024

## Discussion

Our study described the first autochthonous cases of Oropouche fever in Santa Catarina, also the first in the Southern Brazil, an area considered non-endemic for the disease. All cases presented with symptoms similar to those described in the literature, without severe cases or hospitalizations, with half of them reporting recurrence of symptoms. Most interviewees worked in industrial goods and services production, living and working in urban areas, but often close to waterbodies and natural vegetation. 

A study limitation consisted of identifying cases via positive laboratory tests which, in the context of the investigation, was conditioned by the availability of samples previously analyzed for dengue and stored in the sample bank of the Santa Catarina Central Public Health Laboratory. Thus, despite records of individuals with characteristic cases of Oropouche fever in the same period as the confirmed cases, not all had samples collected for laboratory diagnosis, which may have resulted in an underestimation of the outbreak magnitude. Additionally, the interviews were conducted on average 40 days after symptom onset which may have introduced recall bias, compromising data accuracy and influencing both the characterization and identification of symptom recurrence. 

Oropouche fever is an arbovirus caused by a virus of the genus *Orthobunyavirus* (family *Peribunyaviridae*), characterized by presenting a genome with three RNA segments and the capacity for genetic rearrangement. Transmission occurs through the bite of hematophagous females of *Culicoides* sp., main vector described for the disease which proliferates in humid places with organic matter availability. Wild mammals, such as sloths and non-human primates, can be reservoirs, contributing to maintain the virus in the environment ^1^^),(^^15^^),(^^16^.

Few records in the literature describe in detail occupational groups at higher risk of exposure to OROV, with agricultural and forestry workers being frequently mentioned due to their proximity to environments favorable to *Culicoides* midges ^7^^),(^^17^. Our study identified the professional categories most affected in the context of the outbreak investigated. The area’s economic profile may have influenced these findings, considering that some patients had employment contracts in the industrial goods and services production. This result suggests and reinforces that factors related to the characteristics of the place in which these activities are performed may be more relevant for disease transmission than the specific nature of the work performed.

Signs and symptoms observed presented frequencies similar to those described in the literature for Oropouche fever. Potentially severe manifestation such as hemorrhagic and neurological signs were less frequent, corroborating previously recorded findings ^1^^),(^^15^^),(^^18^. No case presented a worsening clinical condition or need for hospitalization, as there were no records of death, pregnant women, and congenital anomalies resulting from vertical transmission, although such outcomes for Oropouche fever have been recently described ^5^^),(^^9^^),(^^18^. The symptomatic period among the interviewed cases was longer than that described in the literature, which indicates approximately seven days, which may have been influenced by recall bias identified during the investigation process ^1^. Approximately half of the interviewed cases reported possible episodes of symptom recurrence, a proportion similar to that described in the literature, which indicates that up to 60% of cases present with a return of symptoms within two weeks following clinical resolution ^1^^),(^^14^. However, the investigation period may also have influenced the identification and characterization of these recurrence patterns, indicating the need for more timely evaluation in future investigations. Although already recognized and described, symptom recurrence in Oropouche fever still lacks elucidation as to the mechanisms involved. This phenomenon may be related to the host’s immune response and to viral persistence or reactivation in the individual, remaining an important gap in understanding the pathophysiology of the disease. 

Overall, the overlap of clinical manifestations between Oropouche fever and other arboviruses-especially dengue-can contribute to underreporting, mistaken final classification, or delay in suspicion and clinical diagnosis, situations that are potentially increased in areas with circulation of other arboviruses ^19^^)-(^^22^. In this context, over half of the respondents reported the presence of other individuals with similar symptoms in their home and/or neighborhood during the same period of illness, suggesting underreporting of the disease in the region investigated. Additionally, it is common for Oropouche fever cases to be initially captured by dengue surveillance, being notified and submitted to laboratory testing for this arbovirus, as occurred in all the cases investigated herein. As such, the Ministry of Health recommends laboratory testing for OROV in samples with negative results for dengue, Zika and chikungunya to increase the sensitivity of the surveillance system and strengthen case detection. Moreover, it establishes the obligation, within the scope of Oropouche fever surveillance, to perform laboratory diagnosis associated with epidemiological investigation to confirm cases related to the disease ^23^.

Regarding the environmental context, although most live and work in urban areas, most of the residences and workplaces are located close to waterbodies, natural vegetation and/or agricultural plantations, areas characterized by the availability of moist organic matter. Banana cultivation was the most frequent among the mentioned agricultural crops, both in the home and workplace environment. According to the literature, the decomposition of leaf litter (organic matter) originating from banana trees provides favorable conditions for vector development. Additionally, the availability of organic matter associated with factors like humidity and temperature can directly influence the density of the *Culicoides* sp. in these areas and, in turn, favor viral transmission ^1^^),(^^24^^),(^^25^. Proximity of human settlements to environmental contexts like these can facilitate contact between humans and the OROV vector ^24^. This contact was evident in this study, since most respondents reported the presence of *Culicoides* sp. in their homes and/or workplaces, and a large percentage mentioned recurring episodes of bites related to it. Knowing the magnitude of Oropouche fever in Santa Catarina was favored, in part, by the recent decentralization of laboratory diagnosis to the federative units. 

However, we could not determine whether OROV was already circulating in the region previously undetected or whether there was a recent introduction, captured in a timely manner by epidemiological surveillance. Nonetheless, the unprecedented detection of autochthonous cases in Santa Catarina may reflect a combination of factors, including: (i) migratory and trade flows from endemic areas ^15^; (ii) possible genetic rearrangements of the virus ^26^; (iii) environmental conditions conducive to vector proliferation ^25^; and (iv) improved sensitivity of health services and surveillance in identifying and qualifying events of relevance to public health. 

Our findings reinforce the need to conduct, in a timely manner, investigations of Oropouche fever cases as close as possible to the symptomatic period, and to perform follow-up after clinical resolution to improve the clinical and laboratory description of symptom recurrence episodes. Strengthening epidemiological and laboratory surveillance can contribute to these goals. Finally, considering the overlap of clinical manifestations between arboviruses, integrating their surveillance, with single notification and standardized laboratory flows adapted to the reality of each territory, can configure a strengthening mechanism, increasing detection and optimizing the response to emergency events and reemergence of arboviruses in Brazil.

## Data Availability

As this is a health surveillance action, access to data used in this study should be requested from the Santa Catarina State Department of Health clicking on the following links: https://www.saude.sc.gov.br/index.php/pt/servicos/esp/anuencia-institucional and https://ouvidoria.sc.gov.br/cidadao/
