# Supplementary material for: First autochthonous cases of Oropouche fever in Southern Brazil: A case series in a non-endemic area, Santa Catarina, 2024
Source: Epidemiol Serv Saude. 2026 Apr 24;35:e20250864. doi: 10.1590/S2237-96222026v35e20250864.en (PMC13686400; doi:10.1590/S2237-96222026v35e20250864.en)
Supplement: Translation

# Translation: Primeiros casos autóctones de febre do Oropouche na Região Sul: série de casos em área não endêmica, Santa Catarina, 2024

**DOI:** 10.1590/S2237-96222026v35e20250864.pt

**Authors:** Renan Novaes Pinto, Benvindo Joãozinho Sá, Ícaro Gonçalves Santos, Vinicius Pereira Feijó, Ivânia da Costa Folster, Renata Zeferino Marques, Leila Catiani Pedrini Eyng, Natália de Andrade Cabral Marchi, Nilceia das Graças Garcia de Souza, Roseleti Martendal, Fernanda Aragona da Costa, Alessandro Igor da Silva Lopes, Morgana de Freitas Caraciolo, Daniel Garkauskas Ramos, Livia Carla Vinhal Frutuoso, Danielle Cristine Castanha da Silva, Aroldo José Borges Carneiro

**Affiliations:** 1 Ministério da Saúde, Programa de Treinamento em Epidemiologia Aplicada aos Serviços do SUS, Brasília, DF, Brasil; 2 Ministério da Saúde, Departamento de Doenças Transmissíveis, Brasília, Distrito Federal, Brasil; 3 Secretaria de Estado da Saúde de Santa Catarina, Florianópolis, Santa Catarina, SC, Brasil; 4 Secretaria Municipal de Saúde de Botuverá, Botuverá, Santa Catarina, SC, Brasil; 5 Secretaria Municipal de Saúde de Brusque, Brusque, Santa Catarina, SC, Brasil; 6 Secretaria Municipal de Saúde de Guabiruba, Guabiruba, Santa Catarina, SC, Brasil; 7 Secretaria Municipal de Saúde de Luiz Alves, Luiz Alves, Santa Catarina, SC, Brasil; 8 Secretaria Municipal de Saúde de Ilhota, Ilhota, Santa Catarina, SC, Brasil

**Keywords:** Infecção por Vírus Oropouche, Infecções por Arbovírus, Surtos de Doenças, Investigação Epidemiológica, Epidemiologia Descritiva

## Abstract

**Objetivo:**

Descrever em tempo, pessoa e lugar os primeiros casos de febre do Oropouche confirmados em Santa Catarina, avaliando sua autoctonia.

**Métodos:**

Estudo descritivo de série de casos, realizado em residentes de cinco municípios de Santa Catarina, detectados laboratorialmente para febre do Oropouche entre 25 de abril e 12 de junho de 2024. Foram consideradas amostras retrospectivas, negativas para dengue e viáveis para análise molecular (RT-PCR). Utilizou-se dados secundários coletados durante a realização de investigações epidemiológicas e de sistemas de informação. As análises foram realizadas através de estatística descritiva. Considerou-se casos autóctones aqueles com ausência de deslocamento 15 dias antes do início dos sintomas.

**Resultados:**

Dos 41 casos elegíveis, 33 participaram do estudo. A maioria era indivíduos do sexo feminino (63,6%), brancos (63,6%) e em idade economicamente ativa (78,8%). Os sintomas mais frequentes foram cefaleia (93,9%), febre (90,9%) e mialgia (81,8%). Cerca de metade relatou recorrência de sintomas após a cura clínica. Grande parte referiu exposição a corpos d’água (57,6%) e áreas com vegetação natural (48,5%), bem como presença frequente do vetor *Culicoides* sp. em domicílios e locais de trabalho. A maioria residia e trabalhava próximo a ambientes propícios à proliferação do vetor, como áreas agrícolas com cultivo de banana. Nenhum caso relatou deslocamento para outras unidades da federação ou ao exterior.

**Conclusão:**

Os casos em Santa Catarina apresentaram perfil clínico-epidemiológico compatível com descrições anteriores. A detecção inédita do vírus Oropouche na região reforça a necessidade de diagnósticos oportunos e vigilância integrada para arboviroses emergentes e reemergentes no Brasil.

**Aspectos éticos**

**Esta pesquisa respeitou os princípios éticos, obtendo os seguintes dados de aprovação:**

Comitê de ética em pesquisa Comissão Nacional de Ética em Pesquisa

Número do parecer 7.665.911

Data de aprovação 28/6/2025

Certificado de apresentação de apreciação ética 88060725.4.0000.0008

Registro do consentimento livre e esclarecido Dispensado.

## Introdução

A febre do Oropouche é uma arbovirose considerada endêmica na região Amazônica, com registros de surtos em outras regiões do Brasil e da América Latina ^1^. Desde 2023, o Brasil apresenta aumento expressivo no número de casos notificados para a doença, principalmente em estados extra-amazônicos ^2^^),(^^3^.

Inicialmente concentrada em estados da Amazônia Legal, a doença expandiu-se rapidamente, entre 2024 e 2025, para áreas consideradas não endêmicas, com ocorrência de surtos explosivos, principalmente nas Regiões Nordeste e Sudeste, em estados como Bahia, Ceará, Rio de Janeiro, Minas Gerais e Espírito Santo ^2^^)-(^^7^.

De acordo com dados públicos do Ministério da Saúde, foram registrados 834 casos em 2023, 13.856 em 2024 e 11.962 até a Semana Epidemiológica 30 de 2025 ^8^.

A infecção pelo vírus Oropouche (OROV) manifesta-se, em geral, de forma leve e autolimitada; contudo, recentemente foi emitido alerta em razão do registro de óbitos associados à doença, assim como a confirmação da possibilidade de transmissão vertical e da ocorrência de casos de anomalias congênitas e óbitos fetais relacionados à infecção ^9^^)-(^^11^.

A ocorrência de surtos explosivos em diferentes regiões do país, incluindo áreas não endêmicas, associada aos novos achados epidemiológicos relacionados a óbitos e transmissão vertical, representa um importante alerta para saúde pública.

Nesse contexto, a detecção inédita do OROV em Santa Catarina, caracterizando o primeiro registro na Região Sul do Brasil, área considerada não endêmica, motivou a realização de uma investigação epidemiológica com o objetivo de descrever os primeiros casos confirmados no estado em tempo, pessoa e lugar, avaliando sua autoctonia. 

## Métodos

### Delineamento do estudo

Trata-se de um estudo descritivo de série de casos, realizado a partir de detecções laboratoriais do vírus Oropouche (OROV), ocorridas entre os dias 25 de abril e 12 de junho de 2024, em residentes de Botuverá, Brusque, Guabiruba, Ilhota e Luiz Alves, municípios que concentravam os casos confirmados, até aquele momento, em Santa Catarina.

### Contexto

No início de 2024, Botuverá, em Santa Catarina, registrou um aumento incomum de casos suspeitos de dengue, cenário epidemiológico atípico em comparação ao histórico do município. No entanto, a maioria das amostras analisadas apresentou resultado laboratorial negativo para dengue, e a investigação entomológica indicou baixa infestação de *Aedes aegypti* no município. Diante desse cenário, somado aos resultados negativos nos diagnósticos para outras doenças investigadas, ao perfil clínico compatível com arboviroses e à identificação de casos de febre do Oropouche, no mesmo período, em áreas não endêmicas para a doença no Brasil ^2^, a hipótese de se tratar de febre do Oropouche passou a ser considerada. A partir da análise laboratorial de cinco amostras de pacientes sintomáticos em fase aguda, em período oportuno para o diagnóstico molecular, foram detectados no dia 25 de abril de 2024 os primeiros três casos positivos para o OROV em Santa Catarina. 

Com base nessas detecções e diante do cenário epidemiológico atípico que estava sendo observado em Botuverá, o Laboratório Central de Saúde Pública de Santa Catarina realizou análises retrospectivas para OROV em amostras provenientes do município que apresentaram resultados negativos para dengue, Zika e chikungunya, armazenadas na instituição. A partir de novas detecções do OROV em análises retrospectivas, essa estratégia de testagem foi ampliada para incluir material clínico proveniente de outros municípios da região. Como resultado, o vírus foi detectado em dezenas de amostras, confirmando a ocorrência de surto no estado durante o primeiro semestre de 2024.

Santa Catarina é uma das 27 unidades federativas do Brasil, localizada na região Sul. O estado possui divisa ao norte com o Paraná e ao sul com o Rio Grande do Sul, sendo banhado pelo oceano Atlântico a leste, e apresenta fronteira internacional com a Argentina a oeste. Tem uma população de 7.610.361 de habitantes, distribuídos em uma área territorial de 95.730,690 km², com um Índice de Desenvolvimento Humano (IDH) de 0,792, classificado como alto ^12^. Os 295 municípios do estado encontram-se distribuídos em oito macrorregiões de saúde e em 17 regiões de saúde ^13^. 

### Participantes

Durante o período avaliado, foram detectados 50 casos de febre do Oropouche em Santa Catarina. Desses, 41 foram considerados elegíveis para o estudo por atenderem aos seguintes critérios: (i) residência em um dos cinco municípios selecionados para o estudo - Botuverá, Brusque, Guabiruba, Ilhota ou Luiz Alves -; (ii) notificação inicial como caso suspeito de dengue; (iii) realização de coleta de amostra em fase aguda para diagnóstico molecular no Laboratório Central de Saúde Pública; e (iv) resultado não detectável para o vírus da dengue e detectável para o OROV.

Foram considerados como casos autóctones de Santa Catarina aqueles em que não foi identificado histórico de deslocamento para outras unidades da federação ou para outros países no período de 15 dias anteriores ao início dos sintomas, considerando o período de incubação da febre do Oropouche estimado entre 3 e 10 dias ^1^^),(^^9^^),(^^14^. 

### Variáveis

O instrumento para coleta de informações, aplicado pelas equipes de investigação epidemiológica dos territórios, contemplou variáveis sociodemográficas (local de residência; sexo; idade; gestação em andamento; raça/cor da pele; escolaridade; ocupação profissional e local de trabalho), clínico-epidemiológicas (data de início dos sintomas; sinais e sintomas; tempo de duração dos sintomas; quadros de recorrência de sintomas; outros sintomáticos na residência ou vizinhança; doença ou condição crônica; deslocamentos realizados no período de 15 dias antes do início dos sintomas e intervalo entre início dos sintomas e o momento da investigação), relacionadas aos hábitos que poderiam favorecer uma exposição à doença (exposição a ambientes com potencial presença de vetores; utilização de medidas/equipamentos de proteção individual ou coletiva; recorrência de picadas por vetores) e relacionadas às características ambientais dos locais de residência e trabalho (zona territorial; proximidade com áreas com presença de corpos hídricos, vegetação natural, cultivo agrícola e desmatamento; tipo de cultivo agrícola reportado; presença de insetos vetores de arboviroses). 

As ocupações profissionais foram classificadas de acordo com os grandes grupos da Classificação Brasileira de Ocupações. Para indivíduos que relataram trabalhar em mais de um local, os dados foram coletados para cada local especificamente.

### Fontes de dados e mensuração

Foram utilizados dados secundários anonimizados, disponibilizados pela Coordenação-Geral de Vigilância de Arboviroses do Ministério da Saúde e pela Secretaria de Estado de Saúde de Santa Catarina, obtidos através de sistemas de informação utilizados pela vigilância - Sinan net e Gerenciador de Ambiente Laboratorial (GAL) - e provenientes de investigações realizadas em campo, registrados de forma padronizada e sistematizados através de um questionário semiestruturado aplicado pelas equipes de vigilância epidemiológica dos territórios envolvidos, como uma ação de vigilância em saúde, com apoio do nível avançado do Programa de Treinamento em Epidemiologia Aplicada aos Serviços do Sistema Único de Saúde (EpiSUS-Avançado).

### Viés

Como forma de minimizar o viés de memória dos participantes das entrevistas, foram utilizadas prioritariamente as informações registradas nos sistemas de informação e registros de atendimento de saúde dos municípios para as variáveis de data de início de sintomas e sinais e sintomas referidos.

### Tamanho do estudo

O estudo foi realizado em 33 casos de febre do Oropouche, entre os 50 detectados em Santa Catarina de 25 de abril a 12 de junho de 2024.

### Métodos estatísticos

Foram utilizadas estatísticas descritivas, com cálculo de medidas de frequência absoluta e relativa, assim como de medidas de tendência central e dispersão, realizadas por meio do Microsoft Excel 365.

O histograma dos casos confirmados de febre do Oropouche foi elaborado a partir da data de início dos sintomas e plotado por semana epidemiológica. Os casos foram categorizados entre aqueles que foram incluídos e não incluídos no estudo.

Os mapas de distribuição espacial dos casos foram elaborados através do QGIS 3.34.4, utilizando dados anonimizados de latitude e longitude para georreferenciamento dos locais de residência e de trabalho e dados vetoriais para camadas de município, estado e país, disponibilizados pelo Instituto Brasileiro de Geografia e Estatística (IBGE), sob a projeção UTM, fuso 22J, datum SIRGAS 2000.

## Resultados

Entre 25 de abril e 12 de junho de 2024, foram detectados 50 casos de febre do Oropouche em Santa Catarina. Desses, 41 atenderam aos critérios de elegibilidade para o estudo e, entre eles, 33 aceitaram participar das entrevistas conduzidas pelas equipes de vigilância epidemiológica locais, sendo, portanto, incluídos na análise, e oito indivíduos recusaram a participação. Os casos incluídos concentraram-se, quanto à data de início dos sintomas, entre as semanas epidemiológicas 11 e 20 de 2024, com picos de casos ocorrendo nas semanas 13 e 19 (Figura 1). 

**Figura 1:** Número de casos confirmados de febre do Oropouche, por semana epidemiológica a partir da data de início de sintomas, de acordo com casos incluídos (n=33) e não incluídos (n=8) no estudo. Santa Catarina, 2024 (n=41)

Dos 33 indivíduos incluídos no estudo, a maioria era residente de Luiz Alves (12; 36,4%) e Botuverá (10; 30,3%). Observou-se predomínio do sexo feminino (21; 63,6%), de raça/cor da pele autodeclarada branca (21; 63,6%) e de indivíduos com idades entre 20 e 59 anos (26; 78,8%). A mediana de idade dos entrevistados foi de 38 anos, com mínimo de 13 e máximo de 66 anos. Não houve identificação de gestantes entre os casos entrevistados. Quanto à escolaridade, predominaram indivíduos com ensino fundamental incompleto (13; 39,4%), seguido por ensino médio incompleto (11; 33,3%), totalizando a maior parte dos entrevistados (24; 72,7%). A maioria dos indivíduos (25; 75,8%) referiu ter ao menos um vínculo de trabalho, sendo predominante a categoria de trabalhadores da produção de bens e serviços industriais (13; 52,0%). Entre os oito que não exerciam atividade profissional, prevaleceram aposentados (4; 50,0%) (Tabela 1).

**Tabela 1:** Características sociodemográficas dos casos de febre do Oropouche. Santa Catarina, 2024 (n=33)

Variável	n (%)
**Município de residência**	
Luiz Alves	12 (36,4)
Botuverá	10 (30,3)
Brusque	5 (15,2)
Ilhota	5 (15,2)
Guabiruba	1 (3,0)
**Sexo**	
Feminino	21 (63,6)
Masculino	12 (36,4)
**Faixa etária (anos)**	
10-14	1 (3,0)
15-19	2 (6,1)
20-29	8 (24,2)
30-39	7 (21,2)
40-49	6 (18,2)
50-59	5 (15,2)
60-69	4 (12,1)
**Raça/cor da pele**	
Branca	21 (63,6)
Parda	10 (30,3)
Amarela	1 (3,0)
Indígena	1 (3,0)
**Escolaridade**	
Ensino fundamental incompleto	13 (39,4)
Ensino fundamental completo	1 (3,0)
Ensino médio incompleto	11 (33,3)
Ensino médio completo	2 (6,1)
Ensino superior incompleto	2 (6,1)
Ensino superior completo ou mais	4 (12,1)
**Ocupação profissional**	
**Com ocupação (Grande grupo da Classificação Brasileira de Ocupações)**	25 (75,8)
Trabalhadores da produção de bens e serviços industriais	13 (52,0)
Trabalhadores dos serviços, vendedores do comercio em lojas e mercados	7 (28,0)
Trabalhadores agropecuários, florestais e da pesca	2 (8,0)
Trabalhadores de serviços administrativos	2 (8,0)
Profissionais das ciências e das artes	1 (4,0)
Membros superiores do poder público e dirigentes de organizações e empresas	1 (4,0)
**Sem ocupação**	8 (24,2)
Aposentado(a)	4 (50,0)
Cuidador(a) do lar	3 (37,5)
Estudante	1 (12,5)

Os sinais e sintomas mais relatados pelos entrevistados foram cefaleia (31; 93,9%), febre (30; 90,9%), mialgia (27; 81,8%), inapetência (26; 78,8%), cansaço/prostração (25; 75,8%) e artralgia (24; 72,7%) (Figura 2). A mediana de duração dos sintomas foi de 14 dias, com mínimo de dois dias e máximo de 37 dias. Dos 33 participantes, 17 (51,5%) relataram apresentar sintomas semelhantes até 15 dias após cura clínica, com cefaleia (11; 64,7%), fraqueza (7; 41,2%) e mialgia (5; 29,4%). Não foi identificado nenhum registro de casos graves.

**Figura 2:** Caracterização dos casos de febre do Oropouche segundo sinais e sintomas. Santa Catarina, 2024 (n=33) ^a^ Perda de consciência; perda de paladar; ardência na pele; tremor nas mãos; confusão; ouvido entupido (autofonia); desidratação e odor na urina.

Destaca-se que 18 (54,5%) dos participantes relataram a ocorrência de pelo menos outro morador sintomático na residência durante o mesmo período de seu adoecimento, e 17 (51,5%) mencionaram conhecimento de algum vizinho com sintomas semelhantes no mesmo período.

Dos 33 participantes, 19 (57,6%) relataram ter alguma doença ou condição crônica, destacando-se hipertensão arterial (8; 42,1%) e doenças respiratórias (4; 21,1%) como as mais frequentemente relatadas.

Todos os deslocamentos mencionados pelos entrevistados, no período de até 15 dias anteriores ao início dos sintomas, ocorreram entre municípios de Santa Catarina, sem registros de viagens para outras unidades da federação ou para o exterior.

Em relação à exposição a ambientes potencialmente relacionados à presença de vetores, 19 (57,6%) dos entrevistados relataram alguma frequência de deslocamento para áreas com presença de corpos d’água. Para áreas com vegetação natural, 16 (48,5%) relataram esse tipo de exposição.

O uso de alguma medida de proteção para prevenir contato com vetores foi relatada por todos os entrevistados. A utilização de roupas compridas foi referida por 29 (87,9%) dos participantes e 28 (84,8%) relataram o uso de repelentes, sendo que 24 (72,7%) utilizavam ambas as medidas.

A recorrência de picadas por maruins foi relatada por 30 (90,9%) dos participantes, enquanto 28 (84,8%) mencionaram recorrência de picadas por mosquitos.

Foram caracterizados, quanto ao ambiente, 33 locais de residência e 27 locais de trabalho. A maior parte das residências situavam-se em áreas urbanas (19; 57,6%), assim como os locais de trabalho (17; 63,0%) (Tabela 2).

**Tabela 2:** Caracterização dos ambientes de residência (n=33) e trabalho (n=27) dos casos de febre do Oropouche. Santa Catarina, 2024

Variável	Ambiente	
	Residência (N=33)	Trabalho (N=27)
	n (%)	n (%)
**Zona territorial**		
Urbana	19 (57,6)	17 (63,0)
Rural	14 (42,4)	10 (37,0)
**Presença de corpos hídricos**		
Sim	27 (81,8)	22 (81,5)
Afluente^a^	21 (77,8)	11 (50,0)
Lago	5 (18,5)	1 (4,5)
Rio	8 (29,6)	12 (54,5)
Tanque	-	1 (4,5)
Não	6 (18,2)	5 (18,5)
**Presença de vegetação natural**		
Sim	30 (90,9)	22 (81,5)
Não	3 (9,1)	5 (18,5)
**Presença de culturas agrícolas**		
Sim	23 (69,7)	14 (51,9)
Aipim	2 (8,7)	-
Arroz	1 (4,3)	1 (7,1)
Banana	13 (56,5)	7 (50,0)
Batata	1 (4,3)	-
Cana-de-açúcar	-	1 (7,1)
Capim	2 (8,7)	1 (7,1)
Eucalipto	1 (4,3)	1 (7,1)
Flores	-	1 (7,1)
Hortaliças	10 (43,5)	5 (35,7)
Milho	4 (17,4)	1 (7,1)
Outras frutas^b^	3 (13,0)	-
Palmeira	3 (13,0)	1 (7,1)
Não	9 (27,3)	12 (44,4)
Não sei	1 (3,0)	1 (3,7)
**Desmatamento (últimos dois anos)**		
Sim	11 (33,3)	3 (11,1)
Não	21 (63,6)	23 (85,2)
Não sei	1 (3,0)	1 (3,7)
**Presença de Culicoides (maruim)**		
Sim	30 (90,9)	23 (85,2)
Não	3 (9,1)	4 (14,8)
**Presença de mosquitos**		
Sim	29 (87,9)	15 (55,6)
Não	4 (12,1)	11 (40,7)
Não sei	-	1 (3,7)

^a^ Córrego, riacho, ribeirão, canal e nascente; ^b^ Laranja, morango e tangerina.

A presença de corpos hídricos (rios, afluentes, lagos e tanques) foi reportada nas proximidades de 27 (81,8%) das residências e em 22 (81,5%) dos locais de trabalho. Os afluentes foram os elementos hídricos mais frequentemente mencionados no entorno das residências, com 21 (77,8%) registros, e os rios foram os mais citados nas proximidades dos locais de trabalho, com 12 (54,5%) relatos. Proximidade com áreas de vegetação natural foi relatada para 30 (90,9%) residências e 22 (81,5%) locais de trabalho (Tabela 2)

O cultivo agrícola foi reportado nas proximidades de 23 (69,7%) residências e 14 (51,9%) locais de trabalho. Em ambos os contextos, a banana foi a cultura mais mencionada (Tabela 2). 

Não foi relatada ocorrência de desmatamento, nos últimos dois anos, nas proximidades de 21 (63,6%) das residências e de 23 (85,2%) dos locais de trabalho (Tabela 2).

A presença do vetor culicoides foi relatada em ambos os ambientes, com 30 (90,9%) registros em residências e 23 (85,2%) em locais de trabalho. Em relação aos mosquitos, foram registrados 29 (87,9%) relatos em residências e 15 (55,6%) em locais de trabalho (Tabela 2).

A distribuição espacial dos locais de residência e de trabalho dos casos indicou proximidade de corpos d’água e/ou locais com cobertura vegetal (Figura 3).

**Figura 3:** Distribuição espacial dos locais de residência (n=33) e de trabalho (n=27) dos casos de febre do Oropouche em Botuverá, Brusque e Guabiruba (A) e em Luiz Alves e Ilhota (B). Santa Catarina, 2024

## Discussão

O presente estudo descreveu os primeiros casos autóctones de febre do Oropouche em Santa Catarina, os primeiros também na Região Sul do Brasil, área considerada não endêmica para a doença. Os casos apresentaram sintomas semelhantes ao descrito na literatura, sem registros de casos graves ou hospitalizações, com metade destes relatando recorrência dos sintomas. A maioria dos entrevistados trabalhava na produção de bens e serviços industriais, residindo e exercendo atividades laborais em área urbana, porém frequentemente próximos a corpos hídricos e vegetação natural. 

Como limitação deste estudo, a identificação dos casos esteve associada à detecção laboratorial, que, no contexto da investigação, foi condicionada à disponibilidade de amostras previamente analisadas para dengue e armazenadas no banco de amostras do Laboratório Central de Saúde Pública de Santa Catarina. Portanto, apesar de registros de indivíduos com quadros característicos de febre do Oropouche no mesmo período dos casos confirmados, nem todos tiveram amostras coletadas para diagnóstico laboratorial, o que pode ter resultado em subestimação da magnitude do surto. Além disso, as entrevistas foram realizadas em média 40 dias após o início dos sintomas, o que pode ter introduzido viés de memória, comprometendo a precisão das informações e influenciando tanto na caracterização quanto na identificação de recorrência dos sintomas. 

A febre do Oropouche é uma arbovirose causada por um vírus do gênero *Orthobunyavirus* (família *Peribunyaviridae*), caracterizado por apresentar um genoma com três segmentos de RNA e capacidade de rearranjo genético. A transmissão ocorre por meio da picada de fêmeas hematófagas de *Culicoides* sp., principal vetor descrito para a doença, que se prolifera em locais úmidos e com disponibilidade de matéria orgânica. Mamíferos silvestres, como preguiças e primatas não humanos, podem atuar como reservatórios, contribuindo para a manutenção do vírus no ambiente ^1^^),(^^15^^),(^^16^.

Há poucos registros na literatura que descrevem detalhadamente grupos ocupacionais com maior risco de exposição ao vírus Oropouche (OROV), sendo frequentemente mencionados trabalhadores agrícolas e florestais, em razão da proximidade com ambientes favoráveis à presença do vetor culicoide ^7^^),(^^17^. O presente estudo permitiu identificar as categorias profissionais mais acometidas no contexto do surto investigado. O perfil econômico da região pode ter influenciado esses achados, considerando que parte dos casos tinha vínculo laboral nos setores de produção de bens e serviços industriais. Esse resultado pode sugerir e reforçar que fatores relacionados às características do local onde se desenvolve essas atividades podem ter maior relevância para a transmissão da doença do que a natureza específica da ocupação exercida.

Os sinais e sintomas observados neste estudo apresentaram frequências semelhantes às descritas na literatura para febre do Oropouche. Manifestações potencialmente relacionadas à gravidade, como sinais hemorrágicos e neurológicos, foram menos frequentes, o que também corrobora achados previamente registrados ^1^^),(^^15^^),(^^18^. Nenhum dos casos apresentou agravamento do quadro clínico, não havendo hospitalização, assim como não houve registro de óbito, identificação de gestantes e anomalias congênitas decorrentes de transmissão vertical, embora tais desfechos para febre do Oropouche tenham sido descritos recentemente ^5^^),(^^9^^),(^^18^. O período sintomático entre os casos entrevistados foi superior ao descrito na literatura, que indica aproximadamente sete dias de sintomas, resultado que pode ter sido influenciado pelo viés de memória identificado no processo de investigação ^1^. Aproximadamente metade dos casos entrevistados relataram possíveis episódios de recorrência dos sintomas da doença, proporção semelhante à descrita na literatura que aponta até 60% dos casos apresentando retorno de sintomas dentro das duas semanas seguintes à resolução clínica ^1^^),(^^14^. No entanto, o momento de realização da investigação também pode ter influenciado na identificação e na caracterização desses quadros de recorrência, indicando a necessidade de avaliação mais oportuna em futuras investigações. Apesar de já reconhecida e descrita, a recorrência de sintomas na febre do Oropouche ainda carece de elucidação quanto aos mecanismos envolvidos. Tal fenômeno pode estar relacionado à resposta imunológica do hospedeiro assim como à persistência ou reativação viral no indivíduo, permanecendo como uma lacuna importante para a compreensão da fisiopatologia da doença. 

De modo geral, a sobreposição de manifestações clínicas entre a febre do Oropouche e outras arboviroses, especialmente a dengue, pode contribuir para subnotificação, classificação final equivocada ou atraso na suspeição e no diagnóstico clínico, situações potencialmente intensificadas em áreas com circulação de outras arboviroses ^19^^)-(^^22^. Nesse contexto, observou-se que mais da metade dos entrevistados relataram a presença de outros indivíduos com sintomas semelhantes na residência e/ou vizinhança durante o mesmo período de adoecimento, o que sugere subdetecção da doença na região investigada. Além disso, é frequente que casos de febre do Oropouche sejam inicialmente captados no âmbito da vigilância de dengue, sendo notificados e submetidos à testagem laboratorial para essa arbovirose, como ocorreu em todos os casos investigados neste estudo. Diante disso, o Ministério da Saúde recomenda a testagem laboratorial para OROV em amostras com resultado negativo para dengue, Zika e chikungunya, com objetivo de ampliar a sensibilidade do sistema de vigilância e fortalecer a detecção de casos. Além disso, estabelece a obrigatoriedade, no âmbito da vigilância da febre do Oropouche, de realização de diagnóstico laboratorial, associado à investigação epidemiológica, para confirmação de casos relacionados à doença ^23^.

Em relação ao contexto ambiental, embora a maioria resida e trabalhe em áreas classificadas como urbanas, observou-se que a maior parte das residências e locais de trabalho encontra-se situada próximas de corpos hídricos, vegetação natural e/ou plantações agrícolas, áreas caracterizadas pela disponibilidade de matéria orgânica úmida. Entre os cultivos agrícolas mencionados, a cultura de banana foi a mais frequente tanto no contexto ambiental das residências quanto dos locais de trabalho. De acordo com a literatura, a decomposição da serrapilheira (matéria orgânica) originada de bananeiras proporciona condições favoráveis para o desenvolvimento do vetor. Além disso, a disponibilidade de matéria orgânica, associada a fatores como umidade e temperatura, podem influenciar diretamente a densidade do vetor *Culicoides* sp. nessas áreas e, por sua vez, favorecer a transmissão viral ^1^^),(^^24^^),(^^25^. A proximidade de ocupações humanas de contexto ambientais como estes pode facilitar o contato entre humanos e o vetor do OROV ^24^. Esse contato pôde ser evidenciado neste estudo, uma vez que a maioria dos entrevistados relatou presença do vetor *Culicoides* sp. em seus domicílios e/ou locais de trabalho, assim como grande parte mencionou episódios recorrentes de picadas relacionadas a ele. Conhecer a magnitude da febre do Oropouche em Santa Catarina foi favorecido, em parte, pela recente descentralização do diagnóstico laboratorial para as unidades federativas. 

Não foi possível determinar, a partir deste estudo, se o OROV já circulava na região com uma subdetecção prévia ou se houve uma introdução recente, captada oportunamente pela vigilância epidemiológica. Ainda assim, a detecção inédita de casos autóctones na região pode refletir uma combinação de fatores, incluindo: (i) fluxos migratórios e comerciais a partir de áreas endêmicas ^15^; (ii) possíveis rearranjos genéticos do vírus ^26^; (iii) condições ambientais propícias à proliferação do vetor ^25^; e (iv) aprimoramento da sensibilidade dos serviços de saúde e da vigilância para a identificação e qualificação de eventos de relevância à saúde pública. 

Os achados reforçam a necessidade de conduzir, de forma oportuna, investigações de casos de febre do Oropouche o mais próximo possível do período sintomático, bem como realizar o acompanhamento após a resolução clínica, visando aprimorar a descrição clínica e laboratorial de episódios de recorrência de sintomas. O fortalecimento da vigilância epidemiológica e laboratorial pode contribuir para essas finalidades. Por fim, considerando a sobreposição de manifestações clínicas entre arboviroses, a integração de suas vigilâncias, com notificação única e fluxos laboratoriais padronizados e adaptados à realidade de cada território, pode configurar um mecanismo de fortalecimento, aumentando a oportunidade de detecção e otimizando a resposta a eventos de emergência e reemergência de arboviroses no Brasil.

## Data Availability

Por se tratar de uma ação de vigilância em saúde, o acesso aos dados utilizados neste estudo deve ser solicitado à Secretaria de Estado da Saúde de Santa Catarina, por meio dos seguintes links: https://www.saude.sc.gov.br/index.php/pt/servicos/esp/anuencia-institucional e https://ouvidoria.sc.gov.br/cidadao/.
